# The contributions of working memory domains and processes to early mathematical knowledge between preschool and first grade

**DOI:** 10.1007/s00426-021-01496-4

**Published:** 2021-03-07

**Authors:** Chiara De Vita, Hiwet Mariam Costa, Carlo Tomasetto, Maria Chiara Passolunghi

**Affiliations:** 1 Department of Life Sciences, Gaetano Kanizsa Psychology Unit, University of Trieste, via Weiss 21, 34128 Trieste, Italy; 2 Department of Psychology, University of Bologna, Bologna, Italy

## Abstract

Working Memory (WM) plays a crucial role in supporting children’s mathematical learning. However, there is no consensus on the relative contributions of different WM domains (i.e., verbal, visuo-spatial, and numerical–verbal) and processes (i.e., low-control and high-control) to mathematical performance, specifically before and after the onset of formal education. This cross-sectional study examined the relations between WM domains and processes and early mathematical knowledge, comparing a group of children in the second year of preschool (*N* = 66) to a group of first graders (*N* = 110). Results of multigroup path analysis showed that whereas visuo-spatial low-control WM significantly predicted early mathematical knowledge only among preschoolers, verbal low-control WM was a significant predictor only among first graders. Instead, the contribution of visuo-spatial high-control WM emerged as significant for both age groups, as well as that of numerical–verbal WM, although the latter to a greater extent among preschoolers. These findings provide new insights into the WM domains and processes most involved in early mathematical knowledge at different developmental stages, with potential implications for the implementation of age-appropriate training interventions targeting specific WM skills before and after the onset of formal education.

## Introduction

Mathematics represents one of the crowning achievements of human societies, and the question of what underlies its development has attracted a lot of research attention. Indeed, mathematical skills underlie attainment in the activities of everyday life as well as play a critical role in predicting educational and financial success, with relevant implications at both the individual and societal level (e.g., Ancker & Kaufman, 2007; Cragg & Gilmore, 2014; Geary et al., 2013; Reyna & Brainerd, 2007).

Working Memory (WM) is a crucial cognitive factor underlying mathematical learning, influencing both the early foundational stages of number knowledge acquisition and the subsequent emergence and development of problem solving skills (Alloway & Alloway, 2010; De Smedt et al., 2009; DeStefano & LeFevre, 2004; Menon, 2016). However, there is still an absence of consensus on the relative contributions of different WM domains (i.e., verbal, visuo-spatial, and numerical–verbal) and processes (i.e., low-control and high-control) to mathematical performance at different developmental stages of mathematical learning.

More in detail, to the best of our knowledge, no study to date has investigated the relations between different WM domains and WM processes, on the one hand, and early mathematical knowledge, on the other, at such an early stage of development, that is before and after the onset of formal education. Moreover, the results of the WM training literature are mixed and inconsistent to date (Kuhn & Holling, 2014; Passolunghi & Costa, 2016; for a review, see Melby-Lervåg & Hulme, 2013). The present study addressed this issue by comparing preschoolers with first-grade children, to shed light on the contribution of specific WM domains and processes to mathematical knowledge before and after the transition to primary school. The results of this investigation could be useful not only to provide new theoretical insights on the relationship between WM and mathematics, but also from an educational point of view, by suggesting directions to develop age-appropriate training interventions aimed at strengthening the cognitive bases of mathematical learning at different ages. On this last point, being able to target specific strengths and weaknesses within a child, depending on his developmental stage, could provide an alternative approach to typical training.

## Working memory and children’s mathematical learning

The relationship between WM and children’s mathematical learning has been widely investigated in the light of Baddeley and Hitch’s multicomponent model (Baddeley, 1986; Baddeley & Hitch, 1974), which refers to WM as a system that allows both temporary storage and manipulation of information (see also Miyake & Shah, 1999). More in detail, this model includes two passive subordinate modality-dependent systems, the phonological loop and the visuo-spatial sketchpad, responsible for short-term storage of verbal and visuo-spatial information, respectively, along with a central executive component involved in coordinating the on-going storage and processing of information in the passive systems, as well as in high-level control, such as task switching, and monitoring allocation of attentional resources (Baddeley, 1986; Cowan, 2008). Therefore, within the Baddeley and Hitch WM model it is possible to distinguish between low-control WM processes used for passively maintaining either verbal or visuo-spatial information and requiring a lower level of attentional control, and high-control WM processes requiring higher levels of attentional control by central executive (see also Cornoldi & Vecchi, 2000, 2003; Cowan, 2008). In the previous literature, high-control WM processes are typically referred to as Working Memory, while low-control WM processes are considered to be representative of Short-Term Memory (STM) (Bull et al., 2008; de Abreu et al., 2010; Gathercole & Alloway, 2006; Giofrè et al., 2013; Mammarella et al., 2008; Passolunghi & Lanfranchi, 2012; Passolunghi & Siegel, 2001; Swanson & Luxenberg, 2009; Unsworth & Engle, 2007b). Especially when working with very young children, as in the case of the present study, both high- and low-control WM measures should be taken into account to achieve a more complete understanding of a child’s WM skills (see Allen et al., 2020a).

Several cross-sectional and longitudinal studies showed a strong relationship between WM skills and mathematical development (e.g., Bull et al., 1999; De Smedt et al., 2009; Geary, 1993; Mazzocco & Kover, 2007; Passolunghi & Siegel, 2001, 2004; Passolunghi et al., 1999; Swanson, 1993). Indeed, even the simplest mathematical calculations require WM abilities, involving both passive (e.g., temporary storage of problem information, retrieval of relevant procedures) and active (e.g., manipulation of quantity representations and task-relevant information, and processing operations to convert them into numerical output) processes (Bisanz et al., 2005; Geary, 2013; Hitch, 1978; LeFevre et al., 2005). However, the nature of the relationship between WM and mathematical learning is likely to vary depending on factors such as task complexity and children’ age and mathematical proficiency (for a review see Raghubar et al., 2010), thus dynamically changing over development (De Smedt et al., 2009; Holmes & Adams, 2006; Klesczewski et al., 2017; McKenzie et al., 2003; Menon, 2016; Raghubar et al., 2010; Rasmussen & Bisanz, 2005).

## Working memory domains and mathematical learning

Although previous research has extensively explored the role of verbal and visuo-spatial WM domains in the development of mathematical learning (for a review see Peng et al., 2016), findings are still inconsistent and inconclusive. More specifically, visuo-spatial WM skills have been found to be strongly related to mathematics not only in preschool years, when children are in the process of acquiring basic number knowledge (e.g., De Smedt et al., 2009; Holmes & Adams, 2006; Kyttälä, Aunio, Letho, Van Luit, & Hautamäki, 2003; McKenzie et al., 2003; Van de Weijer-Bergsma et al., 2015), but also during primary school years, by being involved, for example, in the implementation of written calculation procedures and mental arithmetic (e.g., Allen et al., 2020a; Ashkenazi et al., 2013; Bull et al., 2008; Caviola et al., 2014; Kyttälä & Lehto, 2008; Lee & Kang, 2002; Mammarella et al., 2017; Szűcs et al., 2014; Trbovich & LeFevre, 2003).

Regarding the contributions of the verbal WM domain to the development of mathematical knowledge, findings are especially controversial. Indeed, on the one hand, some studies suggest an increasing involvement of verbal WM skills in mathematical cognition as children grow older (De Smedt et al., 2009; Rasmussen & Bisanz, 2005; Roussel, Fayol, & Barrouillet, 2002; see Friso-Van Den Bos et al., 2013 for a meta-analysis), with specific implications in basic fact retrieval (Holmes & Adams, 2006) and mathematical multiple steps tasks such as calculation (Purpura et al., 2017). On the other hand, however, other research has showed that the contribution of verbal WM is typically more evident during very early stages of mathematical skills acquisition (i.e., ages 4–5), when phonological representations for numbers are still not consolidated and word-based problem solving competence relies more on reading comprehension (Gersten et al., 2005; Martin et al., 2014). Along these lines, visuo-spatial WM skills would play an increasingly critical role during later stages of mathematical learning in building quantity representation and efficiently manipulating it during problem solving, generally enhancing mathematical proficiency (Menon, 2016; Meyer et al., 2010; Soltanlou et al., 2015). As a case in point, Szűcs and colleagues (2014) highlighted strong links between visuo-spatial WM skills, but not verbal WM measures, and mathematical abilities in a large sample of 9-year-old children.

With reference to the verbal WM domain, the ability to memorize and process numerical information seems to play a specific role. Actually, studies conducted on children with Mathematical Learning Disability (MLD) showed that performance on verbal WM tasks involving the processing of numerical information (e.g., digit span tasks) is more frequently related to mathematical difficulties than performance on non-numerical verbal WM tasks (e.g., word span tasks) (e.g., Andersson & Lyxell, 2007; Geary et al., 2007; Hitch & McAuley, 1991; Passolunghi & Cornoldi, 2008; Passolunghi & Siegel, 2001, 2004; Peng & Fuchs, 2016; Peng et al., 2012, 2016). Moreover, a recent research conducted by Allen and colleagues (2020b) in a sample of 7- to 8-year-old typically developing children found that numerical–verbal WM skills appear to be more predictive of mathematical performance than visuo-spatial ones when the two are compared directly.

In line with these results, findings from neuropsychological and behavioural studies provide evidence for a neurobiological disassociation between numerical and verbal processing, thus suggesting that numerical and verbal WM domains might be distinct (Cappelletti et al., 2001). More in detail, the isolation of numerical processing from other cognitive abilities has been showed by various case studies of patients with clinical disorders, such as dementia and aphasia (for an in-depth examination, see Cappelletti et al., 2001), which, in some cases, found preserved numerical knowledge while in others indicated the selective impairment of numerical skills. Considering the neuropsychological and behavioural evidence, several cognitive, anatomic, and functional models have been proposed to describe the functional architecture of number processing systems, also useful for assessing number and calculation disorders (e.g., Cipolotti & Butterworth, 1995; Dehaene & Cohen, 1995; McCloskey et al., 1985). These findings highlight the importance of including numerical measures as part of a cognitive assessment both in typical and atypical development. Specifically, the horizontal segment of the intraparietal sulcus would be mainly activated during tasks involving only numerical information processing (Dehaene et al., 2003). Taken together, these findings suggest that numerical–verbal WM processing skills could be critical in predicting children’s mathematical development (Peng & Fuchs, 2016). However, to our knowledge, research investigating the different role played by numerical and non-numerical verbal WM domains with respect to the development of mathematical skills focused mainly on children with MLD, but not reporting on typically developing children as young as those in the present study (see Allen et al., 2020b for a research on primary school children).

## Working memory processes and mathematical learning

In addition to focusing on WM domains, the literature has also made a distinction between different WM processes (Cornoldi & Vecchi, 2000, 2003; Cowan, 1988, 1995, 2008; Gathercole & Alloway, 2006; Kail & Hall, 2001; Kintsch et al., 1999), suggesting that high-control WM processes requiring concurrent storage, processing, and effortful mental activity, are “active” and entail a main role of attentional control of the central executive component (Gathercole & Pickering, 2000; Passolunghi & Siegel, 2004; Shah & Miyake, 2005). Conversely, low-control WM processes (or STM) refer to a “passive” storage system involved in retaining small amounts of information subsequently retrieved without any manipulation (Cornoldi & Vecchi, 2000, 2003; Engle, 2002). Whereas dual-tasks involving concurrent storage and manipulation of the temporarily held information are traditionally used to assess high-control processes (Cowan, 1995, 2008; Engle, Tuholski, Laughlin, & Conway, 1999; Gathercole & Pickering, 2000; Kane et al., 2004), simple span forward tasks requiring the recall of a sequence of verbal or visuo-spatial information in the same order of presentation are typically used to measure low-control WM processes (Colbert & Bo, 2017; Cornoldi & Vecchi, 2003; Engle, 2002; Unsworth & Engle, 2007b; see also Allen et al., 2020a for a distinction between dual and simple WM tasks).

High- and low-control WM processes are also characterized by different developmental progressions, since the latter develop earlier and faster than the former (see Diamond, 2013). In this regard, previous studies have found that WM performance improves with age as children grow older (Anderson, 2001; Engle et al., 1999; Kail & Salthouse, 1994; Luciana & Nelson, 1998; Tsujimoto et al., 2004; White et al., 2002), showing that WM skills guided by prefrontal cortex (PFC), namely high-control WM processes, emerge at around the age of 4 years and improve substantially between the ages of 5 and 7 years (Anderson, 2001; Luciana & Nelson, 1998), that is the window of time in which children begin to be exposed to formal education.

Previous literature highlights the contribution of high-control WM processes to mathematics both in the early and in the later phases of mathematical learning. In preschool years, high-control WM processes provide scaffolding for building new semantic representations and contribute to emergent foundational mathematical skills (Espy et al., 2004; Passolunghi & Lanfranchi, 2012). At subsequent stages, in primary school, such processes support performance on single-digit addition arithmetic tasks and rule-based arithmetic word problems, through the active maintenance of intermediate results, and foster transitions from more basic (e.g., counting) to more complex (e.g., decomposition) arithmetic procedures and solution strategies (De Smedt et al., 2009; DeStefano & LeFevre, 2004; Geary et al., 2012; Imbo & Vandierendonck, 2007; Menon, 2016; Passolunghi, 2012; Passolunghi & Pazzaglia, 2004; Passolunghi et al., 2007; Swanson, 2006; Swanson & Kim, 2007).

Regarding the link between low-control WM processes and mathematical knowledge, results are more inconsistent. On the one side, some studies did not find a significant relation between low-control WM skills and mathematical achievement in preschoolers (e.g., Passolunghi & Lanfranchi, 2012) or in primary school (e.g., Imbo & Vandierendonck, 2007). On the other side, an involvement of low-control WM skills emerged in counting (Logie & Baddeley, 1987) and calculation procedures requiring the temporary storage of information, but not carrying or borrowing operations (Fürst & Hitch, 2000) as well as in predicting primary school children’s problem solving accuracy (Fung & Swanson, 2017).

Taken together, these patterns of relations suggest that the relative contributions of different WM domains and processes may change dynamically over time depending on children’s age, developmental stage, and expertise. However, it remains unclear what the relative contributions of different WM domains (i.e., verbal, visuo-spatial, and numerical–verbal) and WM processes (i.e., high-control and low-control) to early mathematical knowledge are, specifically before and after the onset of formal education.

## The present study

This study had a twofold aim. First, we sought to explore the contribution of different WM domains (i.e., verbal, visuo-spatial, and numerical–verbal) to early mathematical knowledge at the transition between preschool and primary school. In line with previous studies (e.g., De Smedt et al., 2009; Holmes & Adams, 2006; McKenzie et al., 2003; Rasmussen & Bisanz, 2005), we hypothesized a stronger relation between the visuo-spatial WM domain and mathematics among preschoolers than first graders, with an increasing involvement of the verbal WM domain in first grade. Based on previous studies on both children with MLD (Andersson & Lyxell, 2007; Hitch & McAuley, 1991; Peng & Fuchs, 2016; Peng et al., 2016; Siegel & Ryan, 1989) and typically developing primary school children (see Allen et al., 2020b), we also expected a crucial contribution of the numerical–verbal WM domain in predicting early mathematical knowledge in both preschoolers and first graders, thus remaining stable with age.

Second, we sought to unravel the contribution of different WM processes (i.e., low-control and high-control) to early mathematical knowledge, with the expectation of a greater role of low-control processes among preschoolers, i.e., prior to children’s exposure to formal schooling, and an increasing involvement of high-control processes among first graders as compared to preschoolers. This hypothesis is rooted in evidence on the progressive development of high-control WM processes associated with the PFC that undergoes considerable change throughout early development, especially between the ages of 5 and 7 years (Anderson, 2001; Luciana & Nelson, 1998; White et al., 2002).

## Methods

### Participants

Participants were 176 children (66 preschoolers: *M*_age_ = 51.82 months, *SD* = 3.02, age range 48–54 months, 30 females; 110 first graders: *M*_age_ = 80.09 months, *SD* = 3.68, age range 72–89 months, 57 females), recruited through six schools located in urban areas of northeastern Italy serving middle socioeconomic background families. All children were Caucasian and fluent Italian speakers, and none had a diagnosis for a developmental disorder or reported vision or hearing problems.

### Procedure

Formal consent was provided by the headmasters of the schools involved in the research and from children’s teachers and parents/guardians. Children also gave verbal assent before being assessed. Testing was carried out over a 2-month period, involving the assessment of WM skills and early mathematical knowledge. Each child was tested individually in a quiet room at school without distracting stimuli on two separate sessions lasting approximately 15 min each. The order of administration of the tasks was counterbalanced across participants.

### Measures

*Working memory*. Six tasks were used to assess WM skills.

*Verbal low-control WM*. To assess verbal low-control WM skill, we administered the *word forward recall task* (Lanfranchi et al., 2004). Participants were presented with a series of familiar two-syllable words and required to recall them in the same order as the presentation. There were two word-lists for each span length (from two to five), for a total of eight trials. The child’s answer was considered correct when all items were recalled in the right order (expected range 0–8).

*Verbal high-control WM*. Verbal high-control WM skill was tested using the *verbal dual task* (Lanfranchi et al., 2004, 2012; Lanfranchi, Jerman, & Vianello, 2009a, 2009b). Children were presented with a list of two to five two-syllable words and asked to both remember the first word on the list and tap on the table when the target word *palla* (ball) was pronounced by the researcher. The task had four different levels of difficulty depending on the list’s length (two, three, four, or five words), for a total of eight trials. A score of one was given when both the initial word of the series was remembered correctly and the secondary task (i.e., tapping) was performed (expected range 0–8).

*Numerical–verbal low-control WM*. To measure numerical–verbal low-control WM skill, we used the *digit forward recall task* (from TEMA; Reynolds & Bigler, 1994; see also Lorusso et al., 2011; Lorusso et al., 2006). Children were presented with a series of single digits and required to recall them in the same order as their presentation. The test was composed of 18 trails, 2 for each of the 9 levels of difficulty (2- to 10-digit spans). A score of one was given for each number recalled in the correct position (expected range 0–108).

*Numerical–verbal high-control WM*. Numerical–verbal high-control WM skill was assessed using the *digit backward recall task* (from TEMA; Reynolds & Bigler, 1994; see also Lorusso et al., 2011; Lorusso et al., 2006). Participants were presented with a series of digits and required to recall them in reverse order. The test was composed by 16 trials, 2 for each of the 8 levels of difficulty (2- to 9-digit spans). A score of one was given for each number recalled in the correct position (expected range 0–88).

In both verbal (low- and high-control) and numerical–verbal (low- and high-control) WM tasks, the stimuli were presented verbally.

*Visuo-spatial low-control WM*. To evaluate visuo-spatial low-control WM skill, we used the *pathway forward recall task* (Lanfranchi et al., 2004; Lanfranchi, Carretti, Spanò, & Cornoldi, 2009a, 2009b). Children were shown a path taken by a small frog on a matrix and asked immediately afterwards to recall the pathway by moving the frog from square to square, reproducing the experimenter’s moves. There were four levels of difficulty depending on the number of jumps along the frog’s path and the size of the chessboard (3 × 3 in the first level with two jumps and 4 × 4 in the other levels, with three, four, and five jumps, respectively), for a total of eight trials. The change of the grid size part way through the task was due to the need to increase the difficulty of the test enhancing complexity and articulation of the paths. The 3 × 3 chessboard was used to familiarize the children with the task and then present them with a more complex grid (4 × 4). There was one point awarded for each path recalled correctly (expected range 0–8).

*Visuo-spatial high-control WM*. Visuo-spatial high-control WM skill was tested using the *visuo-spatial dual task* (Lanfranchi et al., 2004, 2009a). Participants were shown a path taken by a small frog on a 4 × 4 matrix containing one red square. Children had to remember the frog’s starting position along each path and they also needed to tap on the table when the frog moved onto the red square. The task had four different levels of difficulty depending on the number of times the frog jumped (i.e., two, three, four, or five), for a total of eight trials. A score of one was given when both the first position of the pathway was remembered correctly and the secondary task (i.e., tapping) was performed (expected range 0–8).

All six WM tasks were administered using a classic self-terminating procedure whereby, starting with the easiest trials, the tasks became progressively more difficult and participants continued until they were not able to correctly perform the two trials of the same level of difficulty.

*Early mathematical knowledge*. To assess early mathematical knowledge, we used the *Early Number Concepts* subtest from the British Ability Scales (BAS3; Eliot & Smith, 2011). This task consisted of 30 items evaluating different aspects of children’s early mathematical competence, such as counting abilities, number concepts, quantitative understanding, and simple arithmetic. The items were scored by awarding one point for a correct answer and no points for a wrong answer, except for item 3 scored by awarding a maximum of six points for a correct answer (expected range 0–35). More in detail, item 3 was composed by two trials both requiring children to count up to 10, in the first case only by reciting, in the second case by pointing with reciting. Each of the two trials was assigned a score depending on the number of correctly counted digits up to 10, up to a maximum of 6 points in total. The subtest was administered using a self-terminating procedure, whereby the task was interrupted after five consecutive errors.

### Data analyses

Univariate descriptive statistics and bivariate correlations among the study variables were calculated using SPSS 25. A multigroup path analysis was then conducted with Mplus 8.3 to compare the patterns of relations between different WM domains and processes and early mathematical knowledge among preschoolers and first graders. Specifically, the multigroup approach allows us to determine whether the contribution of each WM domain (i.e., verbal, visuo-spatial, and numerical–verbal) and process (i.e., low-control and high-control) to mathematical knowledge differs across the two age groups. To address potential violations of multivariate normality assumptions, a robust likelihood estimation with robust standard errors (MLR) was used. The latter allows any ceiling effects in some of the WM measures in both age groups to be handled.

The analysis consisted of three steps. In the first step (Model 1) we estimated a fully variant model in which all the paths linking single high-and low-control WM processes to mathematical knowledge were allowed to freely vary between the two groups. In the second step (Models 2–7) we then estimated a set of partially invariant models, in which we constrained one path at a time to be equal across age groups, and evaluated how such constraints affected model fit. If fit indices remained unchanged, the more constrained model could be retained as equally informative but more parsimonious (and, therefore, preferable) as compared to the baseline model, and the path could be assumed to be invariant across the two age groups. If constraining a path to equality resulted in poorer fit, then the path should be assumed as differing significantly between groups. In case more than one path emerged as invariant across groups from Models 2–7, we would eventually estimate a further partially invariant model (Model 8) in which all the potentially invariant paths were simultaneously fixed to be equal for preschoolers and first graders. Finally, in the third step of analysis (Model 9), we forced all paths to be equal across preschoolers and first graders (i.e., fully invariant model).

To evaluate the goodness of fit of the models, the Satorra–Bentler scaled *χ*^2^ statistic (SB *χ*^2^), Comparative Fit Index (CFI), Root Mean Square Error of Approximation (RMSEA), and Bayesian Information Criterion (BIC) indices were taken into account. Non-significant SBχ^2^ values were retained as indicative of good fit. Values of CFI > 0.95 and > 0.97, and values of RMSEA < 0.08 and < 0.05 are associated with acceptable and good fit, respectively (Schermelleh-Engel et al., 2003). BIC values can be interpreted only comparatively, with lower values suggesting better fit. To assess differences in model fit between the tested models (i.e., fully variant, partially invariant, and fully invariant), significant SBχ^2^ values are indicative of poorer fit of the more restrictive model. the ΔCFI and the ΔRMSEA criterion (Cheung, 2007) were adopted. In addition, we also compared the CFI, RMSEA and BIC values of the more restrictive and the less restrictive models. With sample size up to *N* $\simeq$ 250, a lowering of 0.005 or more in CFI, supplemented by an increase of 0.010 or more in RMSEA, would indicate that the more restrictive model (i.e., the model in which more parameters are fixed to be equal across groups) fits the data significantly less well than the less restrictive model (Cheung, 2007). As to BIC, differences between 0 and 2, 2 and 6, 6 and 10, and higher than 10 points are indicative of weak, positive, strong, and very strong evidence in support of the model with the lowest BIC values (Raftery, 1995).

## Results

Descriptive statistics, reliability measures, and intercorrelations among study variables are presented in Tables 1 and 2, separately for preschoolers and first graders. It should be noticed that, at the bivariate level, all the investigated WM skills were significantly correlated with early mathematical knowledge in both age groups, except for verbal low-control WM in preschoolers and visuo-spatial low-control WM in first graders. In addition, it is worth mentioning that, while age is significantly correlated with preschoolers’ early mathematical knowledge, this significant correlation does not emerge in first graders. This difference among groups may reside in the fact that, with the beginning of the first grade, other factors may be more associated with mathematical performance than age, such as fluid intelligence or quantity and quality of formal education and learning experiences that children have received at school until then (for a meta-analysis Peng et al., 2019).

**Table 1:** Descriptive statistics, reliability measures, and inter-correlations between all variables for pre-schoolers (*n* = 66)

	Descriptive statistics								Zero-order correlations						
	*M*	*SD*		Min	Max	Skewness	Kurtosis	Reliability	2	3	4	5	6	7	8
1. Age (in months)	51.82	3.02		48	54	− 0.13	− 0.99	–	0.15	0.07	0.36**	0.26*	0.23	0.38**	0.44***
2. Verbal low-control WM	4.05	0.92		2	6	0.28	− 0.49	0.88	–	0.16	0.23	0.10	0.57***	0.29*	0.21
3. Verbal high-control WM	1.91	1.73		0	6	0.61	− 0.39	0.84	–	*-*	.37**	0.38**	0.27*	0.32**	0.29*
4. Visuo-spatial low-control WM	4.73	1.57		0	8	− 1.02	2.07	0.79	–	–	–	0.28*	0.24	0.35**	0.49***
5. Visuo-spatial high-control WM	2.52	2.14		0	8	0.53	-0.61	0.81	–	–	–	–	0.23	0.49***	0.35**
6. Numerical–verbal low-control WM	11.20	4.51		2	24	0.59	0.60	0.87	–	–	–	–	–	0.51***	0.49***
7. Numerical–verbal high-control WM	2.39	3.65		0	14	1.58	1.78	0.85	–	–	–	–	–	–	0.68***
8. Early mathematical knowledge	14.52	5.80		2	27	0.09	-0.75	0.84	–	–	–	–	–	–	–

*M* mean, *SD* standard deviation, *Min* minimun, *Max* maximum

*p ≤ .05, **p ≤ .01, ***p ≤ .001

**Table 2:** Descriptive statistics, reliability measures, and inter-correlations between all variables for first graders (*n* = 110)

	Descriptive statistics							Zero-order correlations						
	*M*	*SD*	Min	Max	Skewness	Kurtosis	Reliability	2	3	4	5	6	7	8
1. Age (in months)	80.09	3.68	72	89	0.14	− 0.76	–	0.16	0.14	0.09	0.17	0.13	− 0.04	0.11
2. Verbal low-control WM	5.13	1.12	0	8	− 0.82	3.59	0.89	-	0.26**	0.19*	0.14	0.62***	0.27**	0.46***
3. Verbal high-control WM	2.50	2.22	0	8	0.68	− 0.76	0.86	–	–	0.19*	0.48***	0.26**	0.31***	0.28**
4. Visuo-spatial low-control WM	6.26	1.77	0	8	− 1.74	3.44	0.77	–	–	*-*	0.24*	0.21*	0.31***	0.18
5. Visuo-spatial high-control WM	3.14	2.59	0	8	0.39	− 1.29	0.80	–	–	–	–	0.13	0.26**	0.32***
6. Numerical–verbal low-control WM	22.33	8.11	2	50	0.76	0.80	0.87	–	–	–	–	–	0.36***	0.47***
7. Numerical–verbal high-control WM	10.40	4.67	1	24	0.34	0.16	0.83	–	–	–	–	–	*–*	0.48***
8. Early mathematical knowledge	28.76	3.35	17	35	− 0.79	1.45	0.94	–	–	–	–	–	–	–

*M*  mean, *SD* standard deviation, *Min* minimun, *Max* maximum

*p ≤ 0.05, **p ≤ 0.01, ***p ≤ 0.001

### Multigroup analyses

Fit indices for all the estimated models are reported in Table 3. In the first step of analysis, we estimated a fully variant model (Model 1), in which all the paths were free to vary across age groups. The model was saturated, and was kept as the reference point for model comparisons.

**Table 3:** Fit indices and comparison of multigroup path models with different equality constraints in predicting early mathematical knowledge for preschoolers (*n* = 66) and first graders (*n* = 110)

	Fit indices					Model comparison (vs. Model 1)	
	SBχ2(*df*)	*p*-value	CFI	RMSEA [90%CI]	BIC	ΔCFI	ΔBIC
Fully variant model							
Model 1—all paths free	0.000	1.000	1.000	0.000 [0.000–0.000]	5929.492	–	–
Partially variant models							
Model 2—verbal low-control WM	4.874(1)	.027	0.965	0.210 [0.057–0.411]	5930.345	0.035	+ 0.853
Model 3—verbal high-control WM	0.013(1)	.907	1.000	0.000 [0.000–0.418]	5924.334	0.000	− 5.158
Model 4 – visuo-spatial low-control WM	8.835 (1)	.003	0.928	0.298 [0.141–0.492]	5934.586	0.072	+ 5.094
Model 5—visuo-spatial high-control WM	1.190(1)	.275	0.998	0.046 [0.000–0.292]	5925.417	0.002	− 4.075
Model 6 – numerical–verbal low-control WM	2.625 (1)	.105	0.985	0.136 [0.000–0.348]	5927.309	0.015	− 2.183
Model 7—numerical–verbal high-control WM	12.581(1)	< .001	0.894	0.363 [0.203–0.553]	5933.871	0.106	+ 4.379
Model 8—verbal high-control WM, visuo-spatial high-control WM, and numerical–verbal low-control WM	4.068(3)	.254	0.990	0.064 [0.000–0.201]	5918.179	0.010	− 11.313
Fully invariant model							
Model 9—all constrained	66.288 (6)	< .001	0.457	0.310 [0.245–0.381]	5952.508	0.543	+ 23.016

WM domains and processes reported for each model are constrained to contribute equally to mathematical knowledge in both groups. Values and significance of the Satorra–Bentler (SB) scaled chi-square (SBχ2) and values of Root Mean Square Error of Approximation (RMSEA) for Models 2–9 also represent the fit difference with Model 1. *df* = degrees of freedom; *CFI* Comparative Fit Index, *CI* Confidence interval, *BIC* Bayesian Information Criterion

As evident from Table 3, results for partially invariant models (Models 2–7) vary across WM skills. Constraining to equality across groups the path linking mathematical knowledge to verbal low-control WM (Model 2), visuo-spatial low-control WM (Model 4), and numerical–verbal high-control WM (Model 7) produced significant increases in SBχ^2^ values, and variations in CFI, RMSEA, and BIC values that were all indicative of non-invariance. Therefore, these WM domains and processes should be regarded as contributing to mathematical knowledge to a different extent depending on whether children were in preschool or first grade.

To the contrary, constraining the contribution of verbal high-control WM (Model 3), visuo-spatial high-control WM (Model 5), and numerical–verbal low-control WM (Model 6) to equality across groups did not result in significant increases in SBχ^2^ values. However, inspection of the CFI, RMSEA, and BIC values yielded more nuanced evidence as regards Model 6. In detail, equality constraints lead to a difference that might be indicative of non-invariance in both CFI (i.e., $\ge$0.005) and RMSEA ($\ge$0.010), with a RMSEA value indicating poor fit (0.136). However, RMSEA values may be artificially high in models with small degrees of freedom and relatively low sample size (Kenny, Kaniskan, & McCoach, 2014), as it is in our case. Moreover, the CFI value of Model 6 (0.985) appears to be indicative of very good fit, and a difference in BIC values higher than 2 (i.e., 2.183) provides positive evidence in favour of the more constrained (i.e., more parsimonious) Model 6 as compared to Model 1. For these reasons, we opted to retain the contribution of verbal high-control WM, visuo-spatial high-control WM, and numerical–verbal low-control WM as invariant across groups. Based on these findings, we also estimated a further partially invariant model (Model 8), in which all the three WM domains and processes were simultaneously set as equally contributing to mathematical knowledge for both groups. The resulting model had an excellent fit to the data. Again, although variations in CFI and RMSEA might be suggestive of non-invariance, the SBχ^2^ value was not significantly worse as compared to the saturated fully variant model (*p* = 0.254), and comparison of BIC values provided very strong evidence (i.e., ΔBIC > 10) in favour of the partially invariant Model 8 over Model 1.

In the final step of analysis, we estimated a fully invariant model (Model 9). However, as predicted given the differences that emerged in the previous step of analysis, fit indices for the fully invariant model were not acceptable. The assumption that different WM domains and processes equally contribute to mathematical knowledge among preschoolers and first graders appears, therefore, as not tenable. In sum, results show that a partially invariant model (Model 8) should be retained as the best fitting and more parsimonious representation of the data.

Final estimates from Model 8 are presented in Table 4. In a nutshell, whereas verbal low-control WM emerged as significant predictor among first graders, but not among pre-schoolers, visuo-spatial low-control predicts early mathematical knowledge only among preschoolers, but not among older students. Numerical–verbal high-control WM was found to predict mathematical knowledge in both groups, but to a much larger extent among preschoolers than among first graders. As regards the paths constrained to be equal across groups, the association between numerical–verbal low-control WM and visuo-spatial high-control WM with mathematical emerged as positive and significant for both preschoolers and first graders, whereas the contribution of verbal high-control WM was found to be null in both groups.

**Table 4:** Multigroup path model estimates for paths linking WM domains and processes to early mathematical knowledge for preschoolers (*n* = 66) and first graders (*n* = 110) (Model 8)

	Preschoolers				First graders			
	Estimate (*SE*)	95%CI [lower/upper]	*β*	*p*	Estimate (*SE*)	95%CI [lower/upper]	*β*	*p*
Verbal low-control WM	− 0.404 (0.656)	[− 1.689/0.882]	− 0.064	.538	0.661 (0.302)	[0.069/1.253]	0.220	0.029
**Verbal high-control WM**	− **0.015 (0.110)**	**[**− **0.232/0.201]**	− **0.005**	**.889**	− **0.015 (0.110)**	**[-0.232/0.201]**	− **0.010**	**0.889**
Visuo-spatial low-control WM	1.054 (0.280)	[0.505/1.604]	0.287	< 0.001	− 0.084 (0.146)	[− 0.370/0.202]	− 0.044	0.564
**Visuo-spatial high-control WM**	**0.215 (0.093)**	**[0.033/0.397]**	**0.079**	**.021**	**0.215 (0.093)**	**[0.033/0.397]**	**0.166**	**0.021**
**Numerical–verbal low-control WM**	**0.099 (0.040)**	**[0.021/0.178]**	**0.077**	**.013**	**0.099 (0.040)**	**[0.021/0.178]**	**0.240**	**0.013**
Numerical–verbal high-control WM	0.831 (0.110)	[0.616/1.047]	0.524	< 0.001	0.221 (0.065)	[0.094/0.347]	0.307	0.001
*R*2	0.548			< .001	.406			< 0.001

Bold indicates paths constrained to be equal across age groups

Overall, the partially invariant models accounted for 57.3% of variance for mathematical knowledge among preschoolers, and 40.0% among first graders. A summary of the significant predictors in the two age groups from Model 8 is given in Table 5.

**Table 5:** A summary of the significant predictors in the two age groups from Model 8

Predictors	Preschoolers		First graders
Verbal low-control WM	–	≠	X
Verbal high-control WM	–	=	–
Visuo-spatial low-control WM	X	≠	–
Visuo-spatial high-control WM	X	=	X
Numerical–verbal low-control WM	X	=	X
Numerical–verbal high-control WM	X	>	X

## Discussion

The current study examined the role of WM skills in predicting early mathematical knowledge, by comparing a group of children in the final year of preschool to a group of first graders. More in detail, we investigated the contributions of different WM domains (i.e., verbal, visuo-spatial, and numerical–verbal) and processes (i.e., low-control and high-control) to early mathematics before and after the onset of formal education.

Overall, our results showed a variation in the role of both WM domains and processes in predicting early mathematical knowledge depending on children’s developmental stage. Specifically, in line with our hypothesis, we found that visuo-spatial WM domain was more strongly associated to mathematics in preschoolers than in first graders. In fact, while both low- and high-control visuo-spatial WM skills significantly predicted mathematical performance among preschoolers, only high-control visuo-spatial WM was predictive of early mathematical knowledge among first graders. This result could be attributable to the type of material proposed in task questions, that is, visuo-spatial stimuli. In this regard, while preschoolers may have been relying almost exclusively on visuo-spatial resources, first graders were likely using a mix of strategies, namely a primarily verbal approach supplemented by visuo-spatial representations (see McKenzie et al., 2003; Raghubar et al., 2010). In first grade, visuo-spatial WM skills result indeed predictive only in the case that high-control processes are involved.

Moreover, in line with what was expected, as regards verbal WM domain, low-control verbal WM skill emerged as a significant predictor of mathematics only among first graders, but not among preschoolers. As anticipated, these age-related differences may suggest that children in the second preschool year heavily rely on visuo-spatial representations of number and quantity (e.g., finger counting or number line) when performing mathematical tasks (Hitch et al., 1988; Rasmussen & Bisanz, 2005), while children in first grade may rather rely more on verbal WM strategies (e.g., verbally retrieve arithmetic facts or memorize the associations between mathematical problems and their solutions) (De Smedt et al., 2009; Hecht et al., 2001; Rasmussen & Bisanz, 2005; Roussel et al., 2002; Swanson & Kim, 2007). The fact that only low-control verbal WM (and not high-control) emerges as a significant predictor of first graders’ mathematical knowledge could be due to the specific mathematical domain examined in our study (e.g., performing additions and subtractions) (for the mathematical *domain specificity* explanation see Van de Weijer-Bergsma et al., 2015).

As expected, both low- and high-control numerical–verbal WM skills played a significant role in both age groups, but high-control numerical–verbal WM was found to predict early mathematical knowledge to a much larger extent among preschoolers than among first graders. This finding adds to the existent body of research by providing new insight into the specific contribution of numerical–verbal WM skills. What emerges is that the ability to remember and manipulate numerical information while performing mathematical tasks is crucial not only in primary school (see Allen et al., 2020b) and in children with MLD, as already highlighted by prior research (Andersson & Lyxell, 2007; Hitch & McAuley, 1991; Passolunghi & Cornoldi, 2008; Passolunghi & Siegel, 2004; Peng et al., 2016; Siegel & Ryan, 1989) but also in very young typically developing children, before and after the onset of formal education. This evidence further supports the theory of “domain specificity” of WM (Ericsson & Kintsch, 1995; Peng et al., 2016; Unsworth & Engle, 2007a), according to which the operation of WM depends on the specific domain of knowledge considered. Although we do recognize that in both numerical–verbal WM and mathematical tasks children process the same type of material, that is numerical stimuli, we believe the numerical–verbal WM domain, integrating domain-specific skills, knowledge, and procedures to meet the demands of numerical WM tasks, is very specific and, as such, should be distinguished from the verbal domain (see also Oakhill et al., 2011; Peng et al., 2017).

Concerning the relationship between different WM processes and early mathematical knowledge, our results revealed a significant contribution of both low- (i.e., passive) and high-control (i.e., active) WM skills among both preschoolers and first graders, although with some differences emerging between the two groups. More specifically, visuo-spatial high- and low-control and numerical–verbal high- and low-control WM processes significantly predicted preschoolers’ mathematical performance, while significant predictors of first graders’ early mathematical knowledge were verbal and numerical–verbal low-control and visuo-spatial and numerical–verbal high-control WM processes.

In brief, regarding visuo-spatial WM, in line with what was assumed, findings showed a leading role of high-control WM processes, over that of low-control WM ones, among first graders compared with preschoolers. On the other hand, verbal high-control WM was found to have a null relation with early mathematical knowledge in both age groups. The latter result could be due to the type of task used to assess verbal high-control WM skills (i.e., a verbal dual task) which may still have been too difficult for both preschoolers and first graders (see also Allen et al., 2020a; Sweller, 1994). Finally, concerning numerical–verbal WM, both high- and low-control processes were found to predict mathematical knowledge in both age groups. Taken together, these findings are in line with previous research suggesting the separability of low-control and high-control WM skills as distinct precursors of early mathematical learning (Cowan, 1995; Passolunghi et al., 2007; Shah & Miyake, 2005; Swanson, 2006). These results also reflect the progressive development of high-control WM processes associated with the parallel maturation of PFC between the ages of 5 and 7 years, that is when it starts the exposure to formal education (Anderson, 2001; Engle et al., 1999; Luciana & Nelson, 1998; White et al., 2002).

The present study, however, is not without limitations. Firstly, it provides a cross-sectional perspective on the relations between different WM domains and processes and early mathematical knowledge. A longitudinal design would be needed to dynamically investigate changes in these links in response to children’s cognitive development and level of education. Second, in the current research, we assessed only WM skills and mathematical performance, without taking into account the potential role of either children’s other cognitive abilities such as domain-general intelligence level or other Executive Functions (e.g., inhibition skills) or environmental factors like parents’ education. Furthermore, we did not consider numerical visuo-spatial WM skills in this research, because composite WM tasks used in previous studies were exclusively numerical–verbal WM measures (see Peng et al., 2016). However, considering our results, we can speculate on a plausible distinct role of numerical visuo-spatial WM skills in predicting mathematical learning in both our age groups. Moreover, we acknowledge that the different scoring procedures used in the different WM tasks may have affected the results as giving a point per correct item allows to provide a clearer picture of exactly where the child reaches saturation, whereas absolute scoring procedures do not make it possible.

It should also be noticed that in our review of relevant research literature we compared results across developmental studies from different countries whose native languages are different from Italian. These two points should be kept in mind since in certain countries formal education starts earlier than in Italy as well as the length of the number-words may vary considerably across languages (e.g., Italian versus English), thus leading to a variation of the demands numerical tasks make to children’s WM resources (see Raghubar et al., 2010). We also recognize that we tested early mathematical knowledge through a subtest that simultaneously taps into different aspects of mathematical competence, thus providing a measure of general mathematical achievement. In this respect, future studies might consider introducing more fine-grained measures to assess specific children’s abilities related to early mathematical knowledge (e.g., number line or Approximate Number System tasks) to better account for the complexity of numerical processing and draw a clearer and more complete picture of the relations between WM and different mathematical skills before and after the onset of formal education.

The present study adds new theoretical insights into the knowledge base on the association between WM and early mathematics in two main ways. On the one hand, it employed tasks targeting different WM domains and tapping WM processes characterized by different levels of attentional control. On the other hand, the present study tested competing analytical models to clarify the crucial relations between WM and early mathematical knowledge before and after the onset of formal education. Future research could consider partitioning the variance for mathematical knowledge that our models have accounted for to understand better how the explained variance can be attributed to the different WM domains and processes.

From an educational point of view, our results provide useful suggestions for the implementation of age-appropriate training interventions tapping specific WM skills at different developmental stages. For example, a WM training mainly focused on both low- and high-control visuo-spatial WM processes could be more adequate in the second preschool year, while activities involving primarily the high-control ones might be more appropriate in first grade. Moreover, both low- and high-control numerical–verbal WM skills could represent a fruitful target for training interventions both in preschool and first grade, highlighting the crucial role of processing numerical content from a very early age for the development of mathematical learning.

## Data Availability

Data are available on request to the corresponding author.
